# Lipid-rich Plaques Detected by Near-infrared Spectroscopy Are More Frequently Exposed to High Shear Stress

**DOI:** 10.1007/s12265-020-10072-x

**Published:** 2020-10-09

**Authors:** Eline M. J. Hartman, Giuseppe De Nisco, Annette M. Kok, Ayla Hoogendoorn, Adriaan Coenen, Frits Mastik, Suze-Anne Korteland, Koen Nieman, Frank J. H. Gijsen, Anton F. W. van der Steen, Joost Daemen, Jolanda J. Wentzel

**Affiliations:** 1 Department of Cardiology, Erasmus MC, Rotterdam, The Netherlands; 2 PolitoBIOMed Lab, Department of Mechanical and Aerospace Engineering, Politecnico di Torino, Turin, Italy; 3 Department of Radiology, Erasmus MC, Rotterdam, The Netherlands; 4 Cardiovascular Institute, Stanford University School of Medicine, Stanford, CA USA

**Keywords:** Coronary artery disease, Near-infrared spectroscopy, Intravascular ultrasound, Wall shear stress, Vulnerable plaques, Lipid-rich plaques

## Abstract

High wall shear stress (WSS) and near-infrared spectroscopy (NIRS) detected lipid-rich plaque (LRP) are both known to be associated with plaque destabilization and future adverse cardiovascular events. However, knowledge of spatial co-localization of LRP and high WSS is lacking. This study investigated the co-localization of LRP based on NIRS and high WSS. Fifty-three patients presenting acute coronary syndrome underwent NIRS-intravascular-ultrasound (NIRS-IVUS) imaging of a non-culprit coronary artery. WSS was obtained using WSS profiling in 3D-reconstructions of the coronary arteries based on fusion of IVUS-segmented lumen and CT-derived 3D-centerline. Thirty-eight vessels were available for final analysis and divided into 0.5 mm/45° sectors. LRP sectors, as identified by NIRS, were more often colocalized with high WSS than sectors without LRP. Moreover, there was a dose-dependent relationship between lipid content and high WSS exposure. This study is a first step in understanding the evolution of LRPs to vulnerable plaques.

**Electronic supplementary material:**

The online version of this article (10.1007/s12265-020-10072-x) contains supplementary material, which is available to authorized users.

**Figure:**
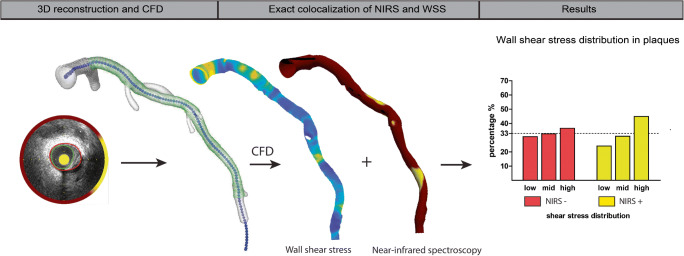
Graphical Abstract

## Introduction

The underlying cause of most cardiovascular diseases is atherosclerosis, an inflammatory-driven disease associated with lipid accumulation in the vessel wall, resulting in plaque formation. The destabilization and rupture of an atherosclerotic plaque remain a leading cause of death. In recent decades, an increasing amount of research has focused on the contribution of local risk factors precipitating future plaque rupture and subsequent cardiovascular events [1–3].

One of these local risk factors is the composition of the plaque. The presence of a lipid pool inside the plaque has been strongly associated with plaque vulnerability, destabilization, and clinical events [3, 4]. Such lipid pools in the vessel wall can currently be detected using the invasive imaging technique near-infrared spectroscopy (NIRS). A combined catheter of NIRS with intravascular ultrasound (IVUS) has been validated for the detection of the local lipid content of coronary plaques [5]. Previous studies using NIRS have shown that the presence of these lipid-rich plaques (LRPs) is independently associated with future major adverse cardiovascular events [3, 4].

A second local factor that is strongly associated with atherosclerosis is wall shear stress (WSS), the frictional force of the bloodstream exerted on the endothelial cells of the vessel wall. Low WSS causes endothelial dysfunction and plays an important role in the development of atherosclerosis [6]. In more advanced stages of atherosclerosis, if plaques intrude into the lumen, WSS will increase. High WSS has been related to apoptosis of smooth muscle cells and consequently to plaque vulnerability and potentially plaque rupture [7]. Recently, high WSS has been proven to be of added value in the prediction of myocardial infarction [8].

Although both high lipid content detected by NIRS and high WSS are associated with plaque destabilization and cardiovascular events, knowledge on co-localization of these two factors is still lacking. We hypothesized that high WSS plays a role in the destabilization of LRPs and thereby contributes to an event. Therefore, in this present study, the co-localization of LRPs and high WSS is investigated in non-culprit segments of patients presenting with an acute coronary syndrome (ACS) as a first step to understand the potential interaction between high WSS and LRPs in plaque destabilization and future events.

## Methods

### Study Design

The IMPACT study was a prospective, single-center study designed to evaluate the association between biomechanical parameters and atherosclerotic disease in non-stented coronary arteries. Hemodynamically stable patients with ACS with at least one non-stented non-culprit coronary segment accessible for intracoronary imaging were eligible for enrollment. Exclusion criteria included the presence of previous coronary artery bypass graft surgery, 3-vessel disease, renal insufficiency (creatinine clearing < 50 ml/min), left ventricular ejection fraction < 30%, and atrial fibrillation. All patients were treated with percutaneous coronary intervention (PCI) of the culprit lesion(s). After successful treatment, a non-culprit segment was imaged according to the IMPACT acquisition protocol. Written informed consent was obtained from all patients. The study protocol was approved by the local medical ethical committee of the Erasmus MC (MEC 2015-535, NL54519.078.15), and the study was conducted in accordance with the World Medical Association Declaration of Helsinki (64th WMA General Assembly, Fortaleza, Brazil, October 2013) and Medical Research Involving Human Subject Act (WMO).

### Data Acquisition

After successful PCI, a non-culprit coronary segment with a length of at least 30 mm and two readily identifiable side branches (diameter > 1.5 mm) was selected as study segment. After angiographic assessment of the non-culprit coronary artery, invasive imaging of the study segment was performed. The images were acquired by an automated pullback (0.5 mm/s) with a NIRS-IVUS catheter (TVC Insight Coronary Imaging Catheter, InfraRedX, Burlington, MA, USA) (0.5 mm/s). Subsequently, invasive local Doppler flow measurements were performed using a ComboWire (Phillips Volcano, Zaventem, Belgium) at different locations between side branches to assess the local blood flow. One month after the invasive measurements, patients visited the outpatient clinic to undergo coronary computed tomography angiography (CCTA) according to standard prospectively ECG-triggered clinical protocol (SOMATOM Force (192 slice 3rd generation dual-source CT scanner), Siemens Healthineers, Germany).

### IVUS-NIRS Image Analysis

Data was anonymized and analyzed offline. As a result of a continuous IVUS pullback, variations in coronary lumen and vessel size due to cardiac motion were present. Therefore, the IVUS images were retrospectively gated by selecting the frame that was located 6 frames before the R-peak using an in-house developed MATLAB (v.2017B, Mathworks Inc, USA) algorithm. The gated frames corresponded with the end-diastolic phase of the cardiac cycle. In all gated NIRS-IVUS frames, the lumen and the external elastic membrane were segmented by an experienced reader (EH). An intra-observer analysis was performed in a random sample of 5 IVUS pullbacks (748 frames) with at least two months interval between the segmentations. A good reproducibility of EEM area, lumen area, and plaque area was found with an interclass correlation coefficient of respectively 0.996 (95%CI 0.996–0.997), 0.983 (95%CI 0.963–0.990), and 0.958 (95%CI 0.939–0.970). In the IVUS images, calcium, defined as a bright signal with a dark shadow behind it, was identified as an angle with the protractor in the center of the lumen. The NIRS signal in the NIRS-IVUS images was analyzed for each degree and labeled as NIRS positive when the signal was > 0.6, implying a high probability for the presence of lipids [9]. The Lipid Core Burden Index (LCBI), the fraction of positive pixels (NIRS pixels positive for lipids/total number of pixels ∗ 1000), was determined for the study segment. Additionally, for each vessel, we identified the 4-mm region with the highest LCBI and thus, the highest lipid content (maxLCBI_4mm_) [10].

### 3D Vessel Reconstruction

By fusing the 3D spatial information of the coronary vessel centerline segmented from the CCTA and the lumen contours extracted from the NIRS-IVUS, a 3D reconstruction was made in MeVisLab (MeVis Medical Solutions AG, Bremen, Germany). The data from the two imaging modalities were matched using large side branches as landmarks, visible in both acquisitions. For subsequent computational fluid dynamics (CFD), reliable inlet and outlets were needed. Therefore, the regions proximal and distal to the IVUS-derived region of interest, as well as side branches (> 1.5 mm), were segmented on the CCTA and scaled and fused with the 3D reconstruction [11]. Of note, for final analysis, only the IVUS-derived region of interest (ROI) was considered. By using a NIRS-IVUS catheter, we could reliably map information on the vessel wall thickness, as well as the NIRS signal onto the ROI of the 3D reconstruction for colocalization analysis.

### Computational Fluid Dynamics

CFD analysis was used to obtain the local wall shear stress according to previously described methodology [12]. In brief, a time-dependent CFD simulation was performed in each reconstructed 3D-geometry, assuming blood as an incompressible, homogeneous, Carreau fluid (Fluent, v.17.1, ANSYS Inc.) [13]. For the CFD simulations, inflow and outflow boundary conditions were required. The flow was derived from intravascular Doppler measurements. The quality of those was examined in a consensus meeting of experts (AH, EH, FG, JW) based on the quality, repeatability, and consistency of the flow signal. For the inflow boundary condition, the most proximal flow measurement of good quality was used as a waveform profile for the time-dependent CFD simulation. Furthermore, for the outflow boundary conditions, the flow distribution through the side branches was calculated based on the intravascular flow measurements at different locations in the coronary artery. For the regions with no reliable flow measures, a previously described scaling law was used to determine the flow ratio between the mother and side branches [14]. To obtain the time-averaged wall shear stress (TAWSS), the computed shear stresses were averaged over a cardiac cycle.

### Data Analysis

**Fig. 1:**
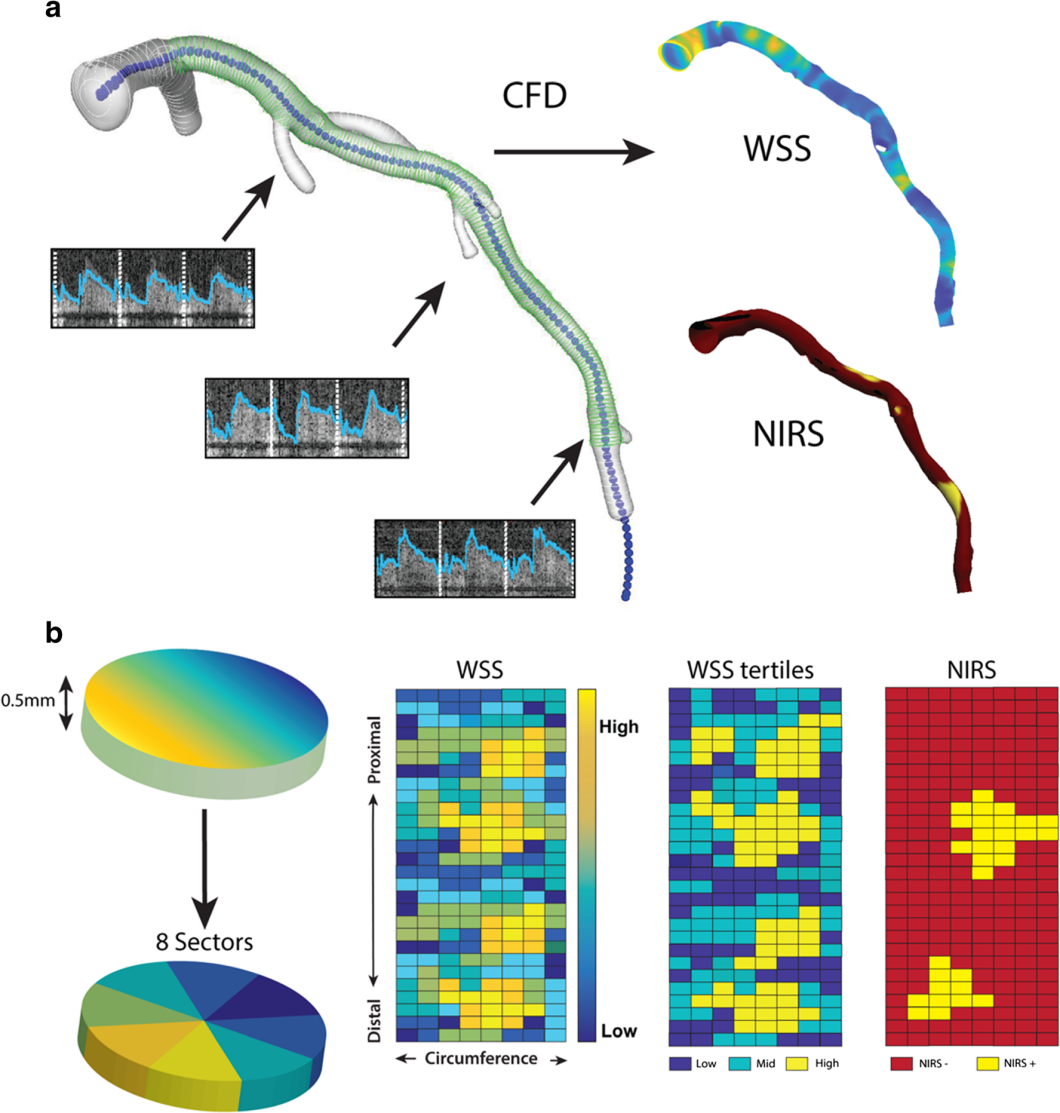
The methodology of the wall shear stress (TAWSS) calculations and analysis of near-infrared spectroscopy (NIRS). **a** IVUS contours (green, the region of interest) and CT contours (white) were matched and fused to make a surface of the 3D reconstruction. At different locations along the artery, flow measurements were done (arrows). Combined, these were used as boundary conditions for the computational fluid dynamics (CFD) and resulted in TAWSS. The NIRS signal was plotted onto the 3D reconstruction for co-localization analysis. **b** The artery was divided into cross-sectional disks of 0.5 mm perpendicular to the centerline of the vessel. These were divided into 8 45° sectors each. A 2D map was created, with on the *x*-axis, the circumference of the vessel, and on the *y*-axis, the distance along the centerline. For TAWSS, the 2D map sectors were divided into low, mid, and high TAWSS. A similar 2D map was created for binary NIRS data (yellow = NIRS positive (NIRS(+)), red = NIRS negative (NIRS(-))

**Fig. 2:**
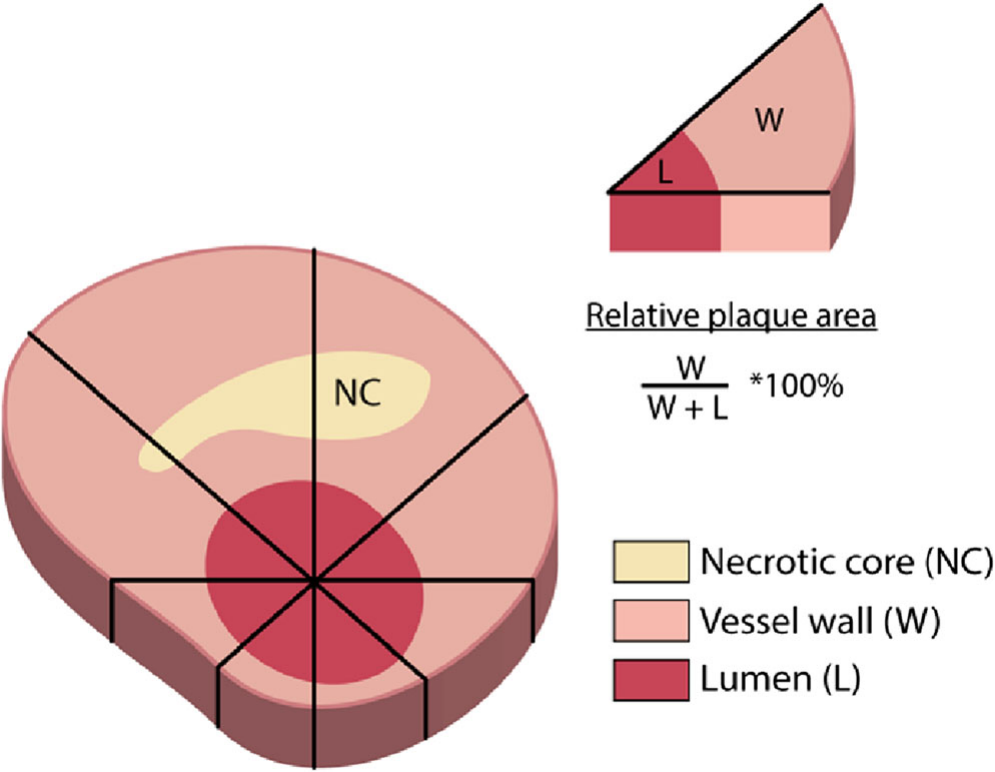
Each cross-section divided into 8 sectors of 45°, based in the middle of the lumen. The relative plaque area was calculated for each sector

All analyzed data was mapped on the 3D geometry of the coronary vessel using VMTK (Orobix, Bergamo, Italy) and MATLAB (v2017b, Mathworks Inc, Natick, MA, USA). For statistical analysis, the 3D reconstruction was converted to a 2D map by folding open the vessel in a longitudinal direction. This type of mapping is comparable to the standard NIRS data display, known as a chemogram. By using this 2D map, all arteries were divided into cross-sectional slices of 0.5 mm thick. All cross-sectional 0.5-mm slices were then divided into 8 angular sectors of 45°, and for all different parameters, the averagevalue per sector was used (Fig. 1). All sectors covering side branches were excluded from further analysis. Additionally, since calcium hampers the visualization of the outer vessel wall, all sectors that contained extensive calcifications (> 90°) were excluded from the analysis. In previous research, a cross-sectional plaque burden was used as a measure for disease burden (plaque area/vessel area ∗ 100). However, in this study, 8 sectors were used for more detailed colocalization. Therefore, a relative plaque area (sectorial plaque area/sectorial vessel area sector ∗ 100%) was calculated (Fig. 2). A sector was regarded NIRS(+) when 50% of data in the sector was NIRS positive (> 0.6 probability of the presence of lipids). For each vessel, TAWSS was divided into vessel-specific tertiles (low, mid, and high), allocating one-third of the sectors in the vessel as high WSS.

### Statistical Analysis

Normally distributed data were shown as mean ± standard deviation. Non-normally distributed data were presented as median (interquartile range (IQR)). For all analyses, sectors with no plaque (wall thickness < 0.5 mm) were excluded. To investigate the statistical significance of the differences in geometrical plaque characteristics between NIRS(+) and NIRS(−) plaque sectors, we used a linear mixed model with NIRS as fixed factor and the individual vessels as random factors to account for within-subject-correlation.

Firstly, a sector-based analysis was performed in the full ROI comparing NIRS(+) plaques and NIRS(−) plaques for differences in geometrical parameters and exposure to TAWSS. Secondly, this analysis was repeated for the sectors that were part of the maxLCBI_4mm_ region, the 4-mm region with the most lipid content. Furthermore, we studied the exposure of high TAWSS in 3 different groups of the maxLCBI_4mm_ based on the LCBI value (< 250, 250–400, and > 400) to research a dose-dependent relationship between LCBI and exposure to high TAWSS. These thresholds were based on previously used thresholds for maxLCBI_4mm_ in studies that showed that patients with higher MaxLCBI_4__mm_ have a higher risk of future adverse cardiac events [3, 4]_._

All differences in TAWSS frequency distributions were assessed using a-χ^2^ test. The differences in wall thickness and relative plaque area were assessed with the Mann–Whitney *U* test. SPSS statistics version 21 for Windows (IBM corp, Armonk, New York) was used for statistical analysis. All tests were 2-tailed, and a *p* < 0.05 was considered significant.

## Results

Between March 2016 and March 2018, a total of 53 patients were enrolled. Four patients withdrew study consent between the baseline procedure and CCTA. There were no major adverse events related to the study procedure. In one patient, it was not possible to match the CCTA-derived centerline with the NIRS-IVUS due to motion artifacts. In one vessel, no NIRS data was acquired during the NIRS-IVUS pullback, and in twelve vessels, less than 1% of the chemogram contained NIRS(+) data. These were all excluded from the analysis. Consequently, 38 vessels from 37 patients were included in the current analysis. Baseline characteristics are listed in Table 1. The mean age of the patients was 62 ± 9 years, 91.9% was male, 24.3% of the patients had a history of previous PCI, and 40.5% of the patients were on statin therapy at the time of inclusion in the study. The studied non-culprit segment was located in the left anterior descending artery in 39% of the cases, in the left circumflex in 29%, and in the right coronary artery in 32% of the cases.

**Table 1:** Baseline characteristics

*N* = 37 patients	
Clinical characteristics	
Age (years)	62 ± 8.9
Men, *n* (%)	34 (91.8%)
Body mass index	27 ± 4.6
Diabetes mellitus, *n* (%)	6 (16.2%)
Hypertension, *n* (%)	13 (35.1%)
Dyslipidemia, *n* (%)	19 (51.4%)
Current smoking, *n* (%)	7 (18.9%)
Positive family history, *n* (%)	16 (43.2%)
Previous MI, *n* (%)	8 (21.6%)
Previous PCI, *n* (%)	9 (24.3%)
LDL (mmol/L)	2.6 (2.1–3.2)
Imaged study vesse	*N* = 38 vessels
LAD, *n* (%)	15 (39%)
LCX, *n* (%)	11 (29%)
RCA, *n* (%)	12 (32%)

### Vessel Characteristics and Relationship to TAWSS

The median length of the IVUS based ROI in the non-culprit segment was 53 mm (43–64). The average TAWSS per vessel ranged from 0.31 to 3.00 Pa, and the median TAWSS over all vessels was 1.16 Pa (0.83–1.61). The median LCBI in the ROI was 54 (35–81), and the maxLCBI_4mm_ was 282 (221–385). Out of the 38 vessels, 29% (*N* = 11) had a maxLCBI_4mm_ > 400.

Dividing the ROI of each vessel into cross-sections of 0.5 mm resulted in a total of 4298 cross-sections. These cross-sections were then divided into 8 sectors of 45°. After removing the sectors with side branches, this resulted in 33,323 sectors of 45°. A total of 1925 (5.8%) sectors were excluded due to the presence of extensive calcifications (> 90°), 20,094 sectors had no plaque (wall thickness < 0.5 mm), and 431 sectors had no reliable NIRS signal. Therefore, 10,873 sectors presenting with plaque but no extensive calcifications were used for further analysis, and 1272 of these sectors were NIRS(+).

**Fig. 3:**
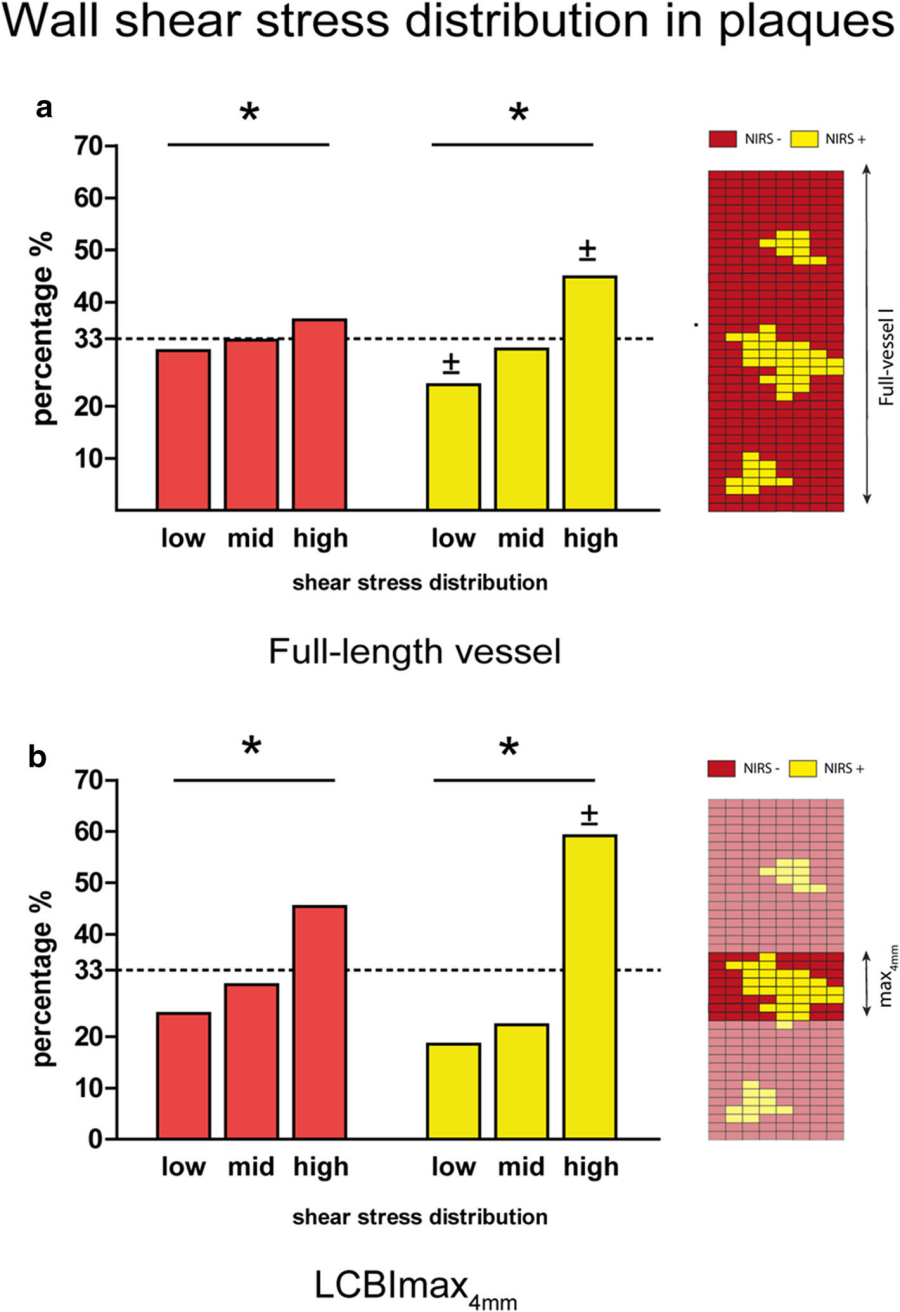
**a** Distribution of the different shear stress tertiles in all sectors with plaque (Wall thickness > 0.5 mm), split into NIRS negative (NIRS(-)) (red) and NIRS positive (NIRS(+)) (yellow) sectors. 2D map showing an exemplary NIRS distribution over the full vessel length. (* *p* < 0.05 for the overall relation, ± *p* < 0.05 compared to the same tertile in NIRS(-) sectors (statistics (χ^2 ^test)). **b** Distribution of the different shear stress tertiles in sectors with plaque (wall thickness > 0.5 mm) in the region with the highest lipid content (maxLCBI_4mm_) split in NIRS(-) (red) and NIRS(+) (yellow) sectors. 2D map showing an example of a NIRS distribution only in the 4 mm with the highest lipid content (maxLCBI_4mm_ ) (* *p* < 0.05 for the overall relation, ± *p* < 0.05 compared to the same tertile in NIRS(-) sectors (statistics: χ^2^ test)).

Table 2 summarizes the plaque characteristics of the sectors that were classified according to their NIRS-derived sectorial lipid status. The wall thickness and the relative plaque area were significantly higher in the NIRS(+) plaque sectors than in the NIRS(−) plaque sectors. Furthermore, the TAWSS distribution between NIRS(+) and NIRS(−) plaque sectors was significantly different. NIRS(−) sectors were for 31% exposed to low, 33% to mid, and 37% to high TAWSS. NIRS(+) sectors were for 24% exposed to low, 31% to mid, and for 45% exposed to high TAWSS (*p* < 0.001) (Fig. 3a). Both for NIRS(−) as for the NIRS(+) sectors, the relative plaque area of the sectors was higher in each subsequent TAWSS tertile (NIRS(−) 48% vs 52% vs 56% and NIRS(+) 54% vs 58% vs 61% *p* < 0.001).

**Table 2:** Plaque characteristics of sectors wall thickness >0.5 mm

	NIRS(−)	NIRS(+)	*p* value
Whole vessel			
Number of sectors	9598	1271	
Wall thickness	0.78 mm (0.75–0.81)	0.88 mm (0.85–0.91)	< 0.001
Relative plaque area	53% (52–55)	58% (56–59)	< 0.001
MaxLCBI_4mm_			
Number of sectors	652	432	
Wall thickness	0.77 mm (0.71–0.83)	0.84 mm (0.78–0.90)	< 0.001
Relative plaque area	53% (50–56)	55% (52–58)	< 0.001

Statistics: Linear mixed model with NIRS status as fixed factor and individual vessel as random factor. Outcome: Estimated means (95% CI for the EM)

**Fig. 4:**
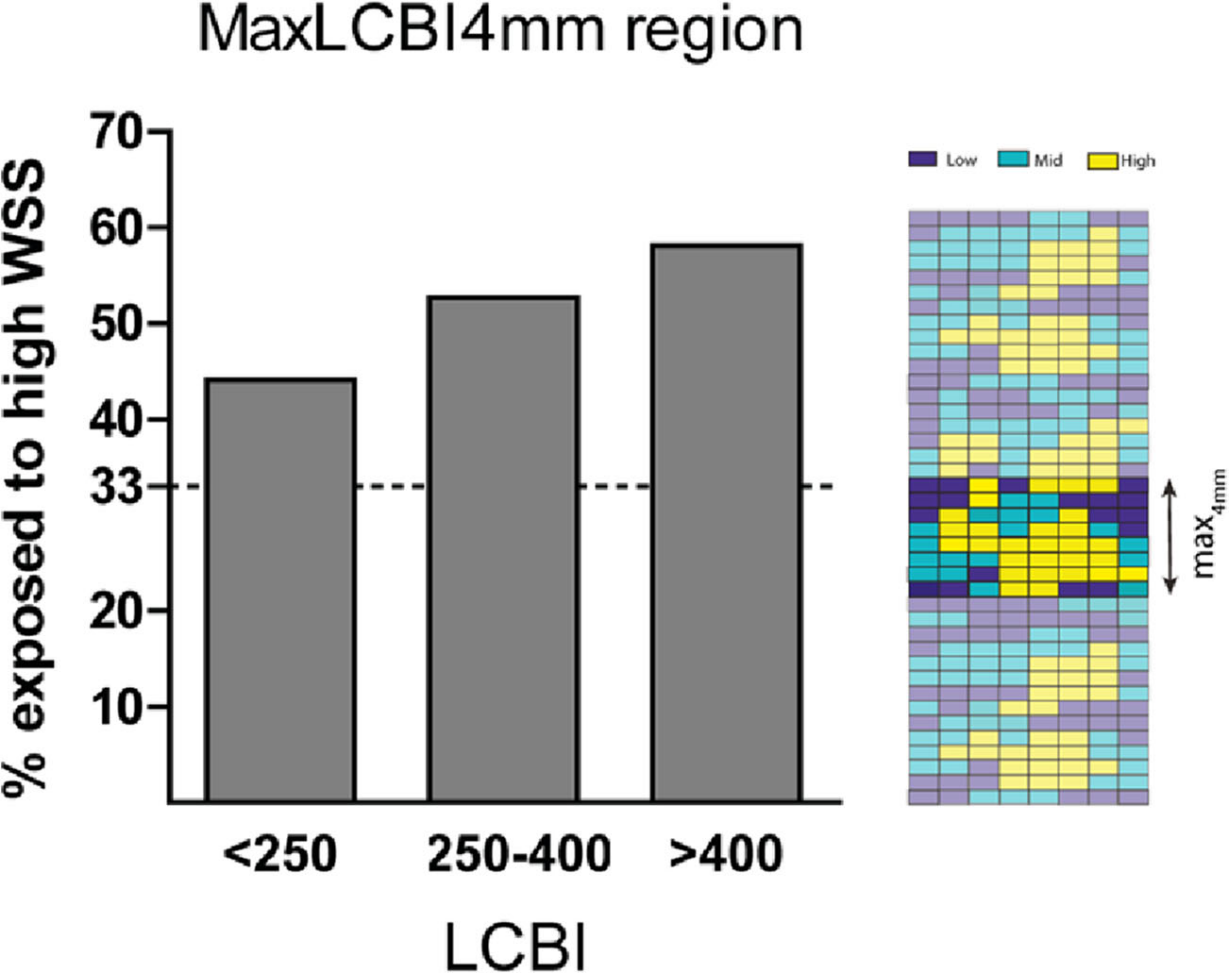
The percentage exposed to high wall shear stress in the maxLCBI_4mm_ regions, split up into 3 groups based on thresholds of maxLCBI_4mm_ (< 250, 250–400, and> 400). 2D map showing an example of an TAWSS tertile distribution in the 4 mm with the highest lipid content (maxLCBI_4mm_ )

In the maxLCBI_4mm_ region, similar to the whole vessel, NIRS(+) sectors were more frequently exposed to high TAWSS than NIRS(−) sectors (59% vs. 45%) (Fig. 3b). When assessing the maxLCBI_4mm_ regions, there was a dose-dependent relation for exposure of high TAWSS and the maxLCBI_4mm_ value. Between low (< 250), mid (250–400), and high (> 400) maxLCBI_4mm_ thresholds, the exposure to high TAWSS in this 4-mm region increased (44% vs. 53% vs. 58%; *p* < 0.001) (Fig. 4). The relative plaque area of the sectors was higher each subsequent maxLCBI_4mm_ threshold (52% vs. 55% vs. 56%; *p* < 0.001).

## Discussion

The present study shows for the first time that LRPs co-localize with exposure to high levels of TAWSS. Additionally, for the most diseased regions in the vessel, as identified by the MaxLCBI_4__mm_, a dose-dependent relation was found between LCBI and exposure to high TAWSS.

LRPs, as detected by NIRS, have been known to be a risk factor for future plaque rupture and major cardiovascular events [3, 4]. Since not all LRPs rupture and result in an event, for clinical decision making and invasive treatment strategies, it is of crucial importance to understand the pathophysiology of LRP in its evolution to high-risk vulnerable plaques. Since plaque destabilization has been proven to be associated with high TAWSS [8], as a first step in unravelling LRP destabilization, we investigated the exposure of LRP to high TAWSS. Therefore, we used detailed invasive imaging with NIRS-IVUS and computational fluid dynamics in non-culprit coronary arteries of acute coronary syndrome patients. In order to discuss the relationship between LRP and high TAWSS, it is essential to understand the interplay between lumen geometry, TAWSS distribution, and the pathophysiology of atherosclerosis. The magnitude of TAWSS is affected by both the flow and the lumen area, such that vessel segments with a smaller lumen but with the same flow are exposed to a higher TAWSS. Low TAWSS, mostly observed at inner curves and near side branches, is associated with plaque initiation and progression [15]. If plaque growth leads to minimal local lumen narrowing, this can already result in a local increase in TAWSS. This increased TAWSS is sensed by the endothelial cells with mechanosensors [16] and initiates compensating outward remodeling of the vessel wall, thereby preserving the lumen area and the TAWSS in the earlier stages of the disease [17]. However, Glagov et al. have shown that if cross-sectional plaque burden (plaque area/vessel area ∗ 100) becomes larger than 40%, lumen narrowing can no longer be prevented, and TAWSS subsequently remains high in these regions [18]. The relative plaque area used in this study is a local surrogate for the cross-sectional plaque burden. The higher relative plaque area found per subsequent TAWSS tertile follows the earlier observations by Glagov that cross-sectional plaque burden and lumen narrowing are linked. Therefore, the observed frequent exposure of NIRS(+) sectors to high TAWSS may be explained by the observed higher relative plaque area of these sectors compared to the NIRS(−) sectors.

Although the higher relative plaque area observed for NIRS(+) sectors might be an obvious explanation for the association between LRP and future events, previous prospective NIRS studies have shown that these relationships were independent of plaque burden, minimal lumen area, or clinical characteristics [3, 4]. Recently, a multimodality imaging study confirmed that segments with higher lipid content as detected by NIRS are colocalized with thin-cap fibroatheromas as visualized with optical coherence tomography [19]. Our finding that the majority of regions with high lipid content colocalize with high TAWSS could therefore be of added value in understanding why LRPs are related to cap thinning and future cardiac events. One of the causes of a coronary event is the rupture of the fibrous cap covering an LRP. High TAWSS is suggested to play an essential role in the thinning of the fibrous cap through apoptosis of smooth muscle cells, thereby increasing the plaque vulnerability and thereby the likelihood of rupture [20]. This hypothesis is supported by previous data of our research group, showing that indeed, plaque ruptures are located in regions with elevated TAWSS [21]. To further support this hypothesis, a clinical study by Kumar et al. demonstrated that higher WSS upstream of coronary lesions is predictive for myocardial infarctions, also implying this relationship between high WSS and plaque rupture [8]. The next step in research would be to combine these findings of colocalization of LRPs and high TAWSS and LRPs and thin-cap fibroatheromas to get more insight into the relationship between high TAWSS exerted on LRPs and the development of a cardiac event. Potentially, this could be done in a retrospective study using previously collected NIRS and event data such as the LRP study [4].

A major advantage of this study was the use of the combined NIRS-IVUS catheter. The dual sensor catheter enabled us to simultaneously and precisely detect the NIRS signal, TAWSS, and wall thickness at a high resolution at the same location. This was due to the following three factors: firstly, the 3D model was based on the coronary lumen derived from the IVUS images; secondly, we could precisely map the NIRS signal on top of IVUS images; and thirdly, no matching step was needed to add the NIRS signal to the model.

In the present study, we used relative TAWSS thresholds based on vessel-specific tertile distribution. This method is in contrast to other studies using absolute TAWSS thresholds [1, 22]. The differences in approach are mainly driven by the different methodologies used to determine TAWSS. Firstly, we used 3D models of all individual coronary arteries, which included all the large side branches of each vessel, whereas other studies used simplified geometrical models without side branches. Although the inclusion of side branches makes the computational model more complex, the side branches are vital to include since they affect the absolute TAWSS values of the main branch. Also, given their unique disturbed flow patterns, bifurcation regions are more prone to plaque formation and should be taken into account in the analysis [23]. Secondly, we used Doppler-derived velocity measurements at multiple locations throughout the artery to obtain patient-specific boundary conditions for the CFD simulations, whereas other studies made more general assumptions regarding temporal flow patterns and flow magnitude. Both differences in the approach have a major influence on absolute TAWSS. In our individually tailored model used here, we observed a very large range in TAWSS among patients. When using absolute thresholds, some patients might present with only sectors labeled as low or high TAWSS. To answer if LRP vs non-LRPs within a patient have a different exposure to high TAWSS, absolute thresholds are not suitable when using our current methodology. Furthermore, we excluded the vessels with no LRPs (LCBI < 10, this is 1% NIRS(+)) to allow the comparison of LRPs and non-LRP coinciding in the same vessel.

## Study Limitations

Firstly, since we aimed at analyzing plaque regions (> 0.5 mm), we excluded all regions with no reliable visualization of EEM, thus also regions with extensive calcifications that block the ultrasound signal. In general, calcified regions are more complex and have thicker plaques and LRPs have shown to colocalize to some extent with extensive calcifications [24]. Therefore, excluding the calcified regions might have led to an overall underestimation of the colocalization of high TAWSS and LRP. If we would assume that calcified regions are most often located in plaques > 0.5 mm, NIRS(+) plaque sectors were even more often exposed to high TAWSS (*p* < 0.05) (see supplement table). Secondly, in the CFD simulations, we assumed rigid walls and the absence of motion of coronary arteries because of cardiac contraction. However, their effect on time-averages TAWSS quantities has been demonstrated to be minor [25].

### Clinical and Future Perspective

Recently, the IBIS-3 trial and the LRP study showed that lipid rich plaques are more at risk for future events [3, 4]. The mechanism that explains the progression of a plaque to an event causing lesion has not been unraveled yet. Since high WSS has been associated with plaque destabilization, LRPs exposed to high WSS could thereby contribute to the event risk of LRPs. Retrospective WSS analysis of the large NIRS studies with clinical follow-up could be the next step in understanding more of the pathophysiological mechanism leading to adverse events.

## Conclusion

In this study, we used detailed invasive imaging and computational fluid dynamics to demonstrate that lipid-rich plaques, as detected by NIRS, are often co-localized with high TAWSS. The co-localization of these two features could be of value in understanding why NIRS(+) plaques are more prone to rupture, leading to clinical events. Future studies are needed to demonstrate the influence of high TAWSS on further lipid-rich plaque development and destabilization.

## Electronic Supplementary Materials

ESM 1 (DOCX 13 kb).

## List of abbreviations

ACS: Acute coronary syndrome
CCTA: Coronary computed tomography angiography
CFD: Computational fluid dynamics
IQR: Interquartile range
IVUS: Intravascular ultrasound
LCBI: Lipid core burden index
LRP: Lipid-rich plaque
MaxLCBI_4__mm_: 4 mm with the highest lipid core burden index in the region of interest
NIRS: Near-infrared spectroscopy
ROI: Region of interest
PCI: Percutaneous coronary intervention
SD: Standard deviation
TAWSS: time-averaged wall shear stress
WSS: Wall shear stress
