# Supplementary material for: Lipid-rich Plaques Detected by Near-infrared Spectroscopy Are More Frequently Exposed to High Shear Stress
Source: J Cardiovasc Transl Res. 2020 Oct 9;14(3):416–25. doi: 10.1007/s12265-020-10072-x (PMC8219563; doi:10.1007/s12265-020-10072-x)
Supplement: Supplementary file 1 — (DOCX 13 kb). [file 12265_2020_10072_MOESM1_ESM.docx]

**Supplement Table**

**Frequency of NIRS(+) and NIRS(-) plaque sectors exposed to low, mid and high time-average wall shear stress**

|  | **Plaque sectors – no calcifications** | | **All plaque sectors** | |
| --- | --- | --- | --- | --- |
| **TAWSS tertile** | **NIRS(-)** | **NIRS(+)** | **NIRS(-)** | **NIRS(+)** |
| **Low** | **31%** | **24%** | **28%** | **22%** |
| **Mid** | **33%** | **31%** | **32%** | **31%** |
| **High** | **37%** | **45%** | **40%** | **47%** |

**Plaque sectors are defined as sectors with wall thickness > 0.5 mm. Plaque sectors – no calcifications are the plaques sectors from which the calcified regions (>90 degrees) are excluded from the analysis; TAWSS: time-average wall shear stress.**
